# Gallbladder cholangiocyte organoids

**DOI:** 10.1111/boc.202400132

**Published:** 2025-02-13

**Authors:** Ankita Dutta, Nandita Chowdhury, Shinjini Chandra, Payel Guha, Vaskar Saha, Dwijit GuhaSarkar

**Affiliations:** ^1^ SOLi3D Laboratory Tata Translational Cancer Research Centre Kolkata India; ^2^ School of Medical Science and Technology Indian Institute of Technology Kharagpur Kharagpur India; ^3^ Department of Paediatric Haematology and Oncology Tata Medical Center Kolkata India; ^4^ Division of Cancer Sciences Faculty of Biology Medicine and Health School of Medical Sciences University of Manchester Manchester UK

**Keywords:** anti‐cancer drug screen, biliary tract cancer, gallbladder cancer, organoids, Wnt pathway

## Abstract

Organoids are miniature three‐dimensional (3D) organ‐like structures developed from primary cells that closely mimic the key histological, functional, and molecular characteristics of their parent organs. These structures self‐organize through cell‐cell and cell‐matrix interaction in culture. In the last decade, organoids and allied 3D culture technologies have catalyzed studies involving developmental biology, disease biology, high‐throughput drug screening, personalized medicine, biomarker discovery, tissue engineering, and regenerative medicine. Many organoid systems have been generated from the gastrointestinal system, for example, intestine, stomach, liver, pancreas, or colon. Gallbladder cancer (GBC) is the most common and highly aggressive form of biliary tract cancer. GBC is rare in the west but has a high incidence in South America and India. Prolonged chronic inflammation is implicated in the pathogenesis of GBC but the driving molecular pathways leading to neoplasia are not well understood. Gallbladder cholangiocyte organoids (GCO) will facilitate the understanding of the evolution of the disease and novel therapeutic strategies. In this review, we have discussed alternative methodologies and culture conditions developed to generate GCO models, applications that these models have been subjected to and the current limitations for the use of GCOs in addressing the challenges in GBC research.

## Graphical abstract

Gallbladder cholangiocyte organoids (GCOs) can be instrumental in investigating gallbladder disease biology and unravelling the molecular mechanism underlying gallbladder cancer (GBC) pathogenesis. Other potential applications of GCOs involve their use in regenerative medicine for cholangiopathies and screening of alternative therapeutics for treating GBC. This article reviews different methodologies and key components employed to develop GCOs for diverse applications; identifies the current limitations, and proposes possible ways to address them.

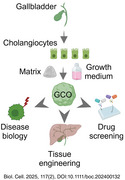

## INTRODUCTION

### Background

For cancer, cell lines and model organisms have made a significant contribution to our understanding of pathogenesis and the development of suitable therapeutic strategies for patients. Cell lines are the most common and simplest models. They are easy to handle, cost‐effective, and can be scaled up for high‐throughput experiments. These artificially immortalized two‐dimensional (2D) systems have limited physiological relevance. Three‐dimensional (3D) spheroid cultures developed using cell lines lack the cell‐matrix interaction. Moreover, cell lines are prone to heterogeneity as mutations are induced during various culture methods. This leads to problems with reproducibility as globally laboratories are likely to be investigating different clones of a single cell line. Furthermore, the generation of primary cell lines from patients is inefficient and requires adaption and selection in 2D culture conditions. Transgenic and patient‐derived xenografts (PDXs) are the most common in vivo models. Transgenic mice are genetically engineered for spontaneous tumor generation. While these mice allow investigation into the genetic and biological evolution of human disease, they are not useful in developing clinical models of disease. PDXs generated by transplanting cancer cells into immunocompromised mice are extensively used but do not accurately reflect tumor development. Engrafted tumors in PDXs do not always show growth kinetics and metastatic properties seen in patients. High‐throughput studies are not feasible and so far, they have had little impact on cancer drug discovery.

An ideal model should be derived from primary patient cells and self‐organize into a 3D structure, recapitulating the genotype and phenotype of the organ of origin and preserve the primary tumor heterogeneity. These models need to be suitable for genetic modification and enable patient‐specific drug testing.

### Organoids

The recent development of *ex vivo* models where primary tissue obtained from patients can be induced to form 3D structures called organoids, shows great promise (Clevers, 2016). An organoid is defined as a 3D structure derived from pluripotent stem cells, or differentiated cells, that self‐organize through cell–cell and cell–matrix interactions, to recapitulate the key aspects of native tissue architecture and function *ex vivo* (Marsee et al., 2021). Organoids closely mimic the key characteristics of the source tissue in a less time‐consuming and cost‐effective manner compared to the animal models. Additionally, the organoids can be scaled up for high‐throughput experiments and are reproducible.

Prior to the advent of organoids, another type of 3D culture known as spheroids were developed from the cell lines, primary cells or tumor biopsies using similar methods as the organoids but in the absence of the extracellular matrix (Thorsen et al., 2007). Spheroids, usually grown in the ultralow attachment plates to prevent cell attachment to the plastic surface, would form 3D structures which lacked any systematic organization in the absence of any cell‐matrix interaction. Unlike the spheroids, which are mere cellular aggregates, organoids are self‐organizing organotypic structures that maintain cellular polarity based on cell‐cell and cell‐matrix interactions and thus bear superior physiological relevance to the source tissues.

To date, organoids have been successfully generated from different organs both healthy and tumor across different species. The key to organoid development is the ability of stem cells, when cultured in ideal conditions, to develop structures that resemble the tissue of origin. Organoids are initiated from two main types of stem cells. The first are the pluripotent embryonic stem cell (ESC) or where adult cells are artificially programmed for pluripotency, that is, the induced pluripotent stem cell (iPSC). The second are the tissue restricted or adult stem cells (ASC) which retain the “memory” of the tissue of origin and self‐organize to develop organoids with tissue‐specific cell types and function. Conditions initially developed to generate ASC tissue specific organoids have now been successfully adapted to generate organoids from cancers originating from the same organs. This advance allows patient‐derived cancer organoids to be used in high throughput drug screening to personalize therapy in a timely fashion. Organoids can be expanded, maintained for long‐term in culture conditions, and cryopreserved for future use. These phenotypically and genetically stable organoids have augmented studies involving developmental biology, disease biology, high throughput drug screening, personalized medicine, biomarker discovery, tissue engineering, and regenerative medicine (Online Resource 1).

### Gallbladder

**FIGURE 1:**
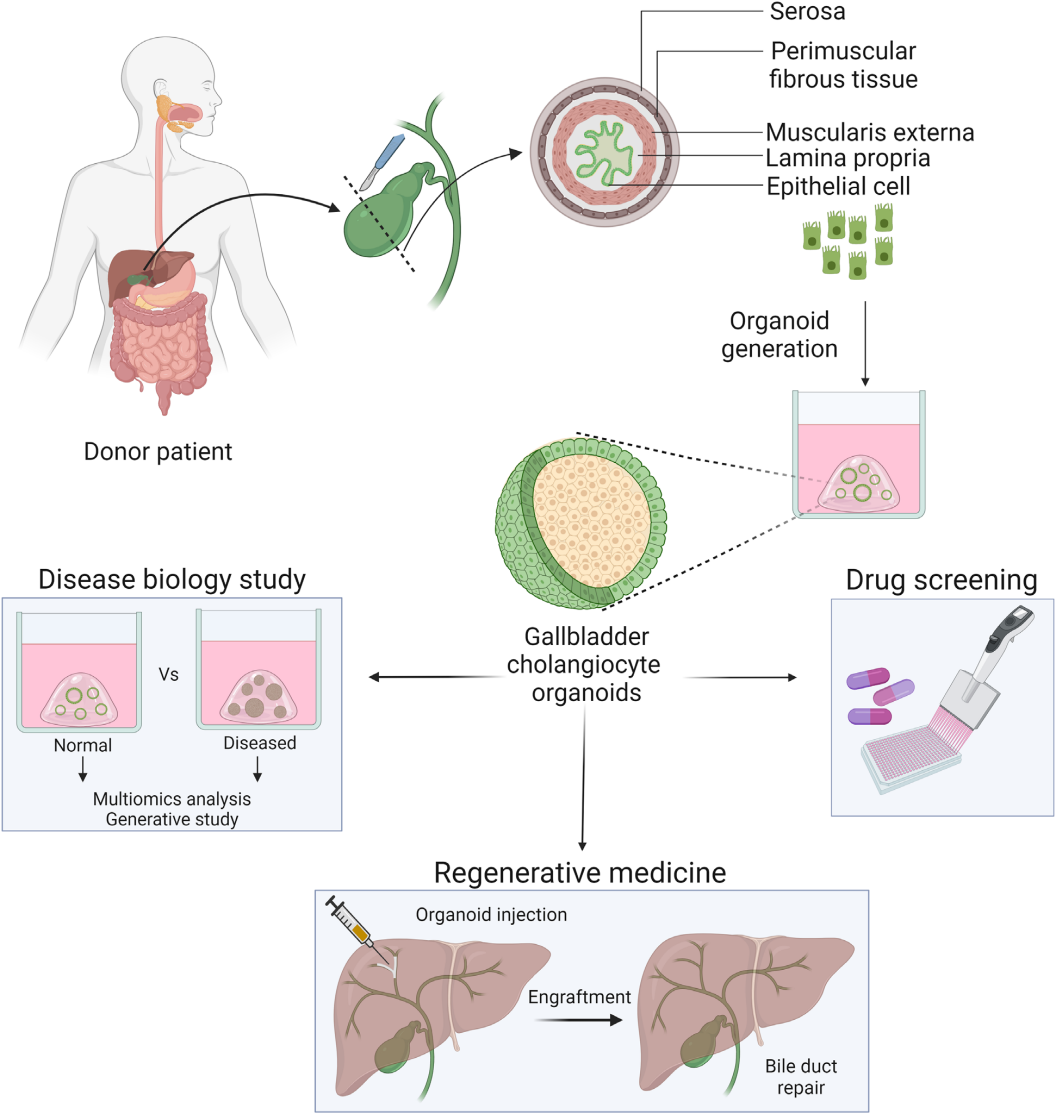
**Establishment of gallbladder cholangiocyte organoids and their potential applications**. Gallbladder cholangiocytes (epithelial cells) are isolated from the mucosal layer of surgically resected gallbladder using mechanical scraping or enzymatic digestion. The cells are washed, passed through strainer and seeded into extracellular matrix domes. The domes are then overlaid with organoid growth media and incubated at 37°C temperature inside a CO2‐incubator for organoid development. Developed organoids can be subjected to various applications, such as gallbladder disease biology study, drug screening and biliary repair and regeneration. Created with BioRender.com.

The biliary tract comprises the intra‐hepatic bile ducts (IHBD), extra‐hepatic bile ducts (EHBD), and the gallbladder. The gallbladder is a sac‐like luminal organ located in the abdomen between the hepatic lobes. It has three distinct anatomical parts—the fundus, the body, and the infundibulum that ends in the cystic duct. The cystic duct joins the hepatic duct and the pancreatic duct to form the common bile duct (CBD), which then enters into the duodenal wall through the Sphincter of Oddi. The histological architecture of the gallbladder consists of the mucosal layer comprising epithelial cells and lamina propria, the smooth muscle layer and the serosa (Roa et al., 2022) (Figure 1). The mucosal layer comprises single columnar epithelial cells (cholangiocytes) expressing cytokeratins and mucins. As compared to the EHBD, cholangiocytes of IHBD expresses significantly increased expression of *PROM1, SOX9*, and *ALB* (Verstegen et al., 2020). No unique markers for gallbladder cholangiocytes have been identified so far.

The liver secretes bile which then flows through the bile ducts and gets collected in the gallbladder. Bile is an alkaline fluid that contains 95% water, electrolytes, and organic molecules like bilirubin, biliverdin, cholesterol, bile acids, and phospholipids. During the digestive phases, the gallbladder contracts under the neuronal stimulus and cholecystokinin (CCK) to release the bile into duodenum that helps in the digestion and absorption of fats and lipids. Secretin also helps to release bile in the intestine when there is an increase in acid reflux.

The transmembrane channel proteins in the gallbladder concentrates the bile and reduces the bile volume by 80%–90%. Bile salt export pump (BSEP) is responsible for the secretion of the bile salts. Cystic fibrosis transmembrane conductance regulator pump (CFTR), a member of ATP‐binding cassette (ABC) is located on the apical side and actively regulates the chloride and water transport in an ATP dependent manner. Multidrug resistant pump1 (MDR1) co‐localized with CFTR pump at the luminal side effluxes the hydrophobic organic molecules to the lumen in ATP dependent manner, thus protecting the gallbladder epithelial cells from continuous exposure to toxic organic compounds. Changes in digestive and absorptive functions of the gallbladder lead to various diseased conditions like inflammation, gallstones and gallbladder cancer.

### Gallbladder Cancer

Gallbladder cancer (GBC) is the most common biliary tract malignancy that arises from gallbladder epithelial cells. GBC is rare in most parts of the world but has a higher incidence in Latin America, parts of Asia and North Africa (GLOBOCAN 2022, https://gco.iarc.fr/en). In India, GBC is more common in the north, east, and north–eastern regions (incidence 10–22 per 100,000) (Dutta et al., 2019), compared to the rest of the country. GBC is more common in females compared to males (2:1 ratio). Symptoms are often non‐specific and patients present with advanced‐stage disease. Treatment involves surgical excision where possible along with an adjuvant or neoadjuvant chemotherapy. If the tumor is inoperable, radiation therapy often follows chemotherapy. Despite providing the current available treatments, outcomes remain poor (median survival less than 10 months (Dutta et al., 2023)).

Pathogenesis of gallbladder cancer appears to be multifactorial. Etiological factors include chronic infection (such as *Salmonella* species), chronic heavy metal poisoning, dietary and life style habits, high parity, family history and genetic factors, gallstones, and obesity (Sharma et al., 2017). The most likely cause is a chronic inflammatory process which over time results in DNA damage and pathogenic mutations (Roa et al., 2022). For most cancers, our understanding of pathogenesis and biology has come from detailed analyses of cell lines and animal models. There are only a few gallbladder cancer cell lines (Ghosh et al., 2004) of Chinese and Japanese ethnicity, and they are not widely available. Murine transgenic (Wu et al., 2007) or PDXs (Kato et al., 2021) are also limited. The lack of suitable laboratory models for GBC has hampered progress in understanding why patients develop this cancer and in identifying better therapies.

In this review, we discuss various methods used by different research groups to develop cholangiocyte organoid models of gallbladder diseases and their applications (Figure 1). Finally, we also note the current limitations of these models along with the future scopes for the use of gallbladder cholangiocyte organoids (GCOs) in addressing the challenges in GBC research.

## DEVELOPMENT OF GALLBLADDER CHOLANGIOCYTE‐DERIVED ORGANOIDS (GCOs)

Organoids were first generated from intestinal epithelium. Intestinal crypt cells, containing leucine‐rich repeat‐containing G protein‐coupled receptor 5 positive (LGR5^+^) intestinal stem cells were grown in conditions that mimic the intestinal crypt. Wnt signaling required for proliferation was achieved by the addition of the Wnt agonist R‐spondin. Epidermal growth factor (EGF) and Noggin were added to induce expansion. Cells were cultured by placing on laminin rich Engelbreth–Holm–Swarm‐based hydrogel (Matrigel) as a source of extracellular matrix. LGR5^+^ cells self‐organized to form organotypic and proliferative crypt‐like structures (Sato et al., 2009). These structures were able to undergo further differentiation to form the villus‐like structures. The same basic principle has been used to generate epithelial organoids from multiple organs with some tissue specific modifications in culture conditions.

Key factors that are required to establish organoids from different tissues include tissue specific growth factors (Clevers, 2016), the matrix used to support the cells and the primary cells from the respective tissues. Critical to this is the regulation of stem cells by the Wnt/β‐Catenin pathway, which has been discussed below with particular reference to GCOs.

### Growth factors

While organoids are successfully developed from ESCs, PSCs, iPSCs, or ASCs and mature epithelial cells, tissues from different sources require specific growth factors. Enrichment of any specific cell populations having either mature epithelial cell properties (e.g., planar cell polarity) or stem‐like properties (e.g., self‐renewal) within the organoids can be modulated by altering one of the key signaling pathways involved in organ development, that is, the Wnt signaling pathway.

#### Wnt pathway (Figure 2)

Wnt signaling plays a pivotal role in the embryonic developmental phases by regulating organ formation, tissue morphogenesis, cell fate determination, and cell polarity. In cancer, aberrant Wnt activation is associated with signaling, disease development, and therapeutic modalities. During organoid development, the primary objective is to stimulate the tissue‐resident stem cells to develop into self‐organizing structures mimicking the parent adult organ, for which Wnt signaling is a key regulator (Nusse & Clevers, 2017).

##### Canonical Wnt pathway

**FIGURE 2:**
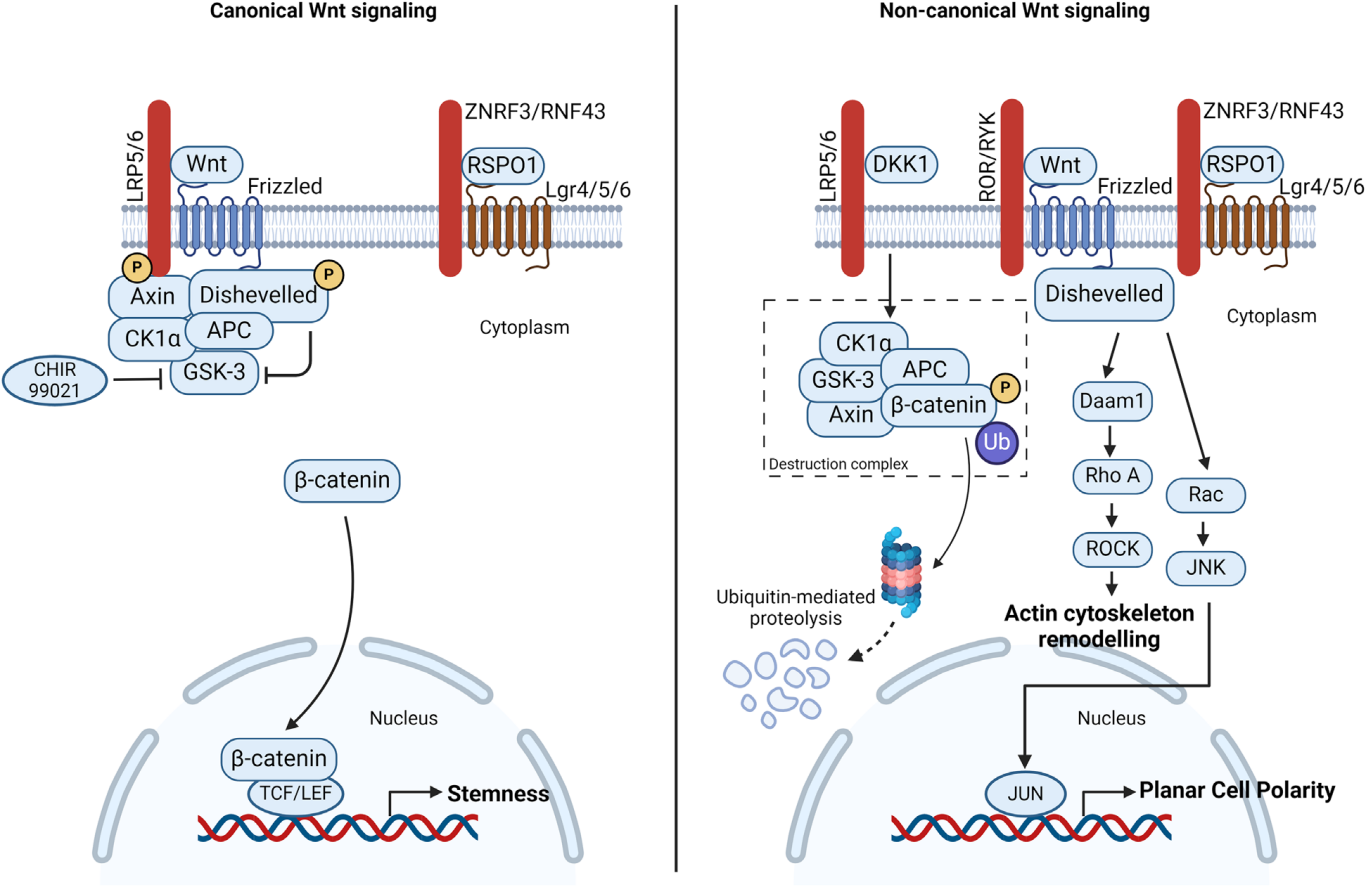
**Overview of the canonical and non‐canonical Wnt signaling pathway**. In the canonical Wnt pathway; binding of WNT ligand to Frizzled and LRP5/6 receptors phosphorylates Dishevelled. This inhibits GSK‐3 kinase activity and prevents destruction complex (APC‐Axin‐GSK3‐CK1α) formation. This stabilizes β‐catenin in the cytosol. Unphosphorylated β‐catenin translocates to the nucleus, where it acts as co‐transcription factor with TCF/LEF to promote transcription of effectors for self‐renewal. RSPO1 binds with ZNRF3/RNF43 and forms a complex with LGR4/5/6 and stabilizes Frizzled. Alternatively, exogenous addition of GSK‐3 inhibitor (CHIR99021) can activate the canonical Wnt pathway. DKK1 inhibits β‐catenin‐dependent Wnt signaling pathway by binding to the LRP5/6 receptor. Competitive binding of DKK1, facilitates WNT binding to Frizzled and activates the non‐canonical Wnt pathway through Ror/Ryk receptor complex. This activates RhoA‐ROCK and JNK pathway. They regulate the cytoskeleton remodelling and planar cell polarity, respectively (Corda & Sala, 2017). DKK, Dickkopf‐1; RSPO1, Rspondin 1; ZNRF3/RNF43, zing and ring finger 3/ ring finger 43; LGR5, leucine‐rich‐repeat‐containing G coupled receptors 4/5/6; TCF, T‐cell factor; LEF, lymphoid enhancer factor; ROR/RYK, retinoic acid‐related orphan receptors/receptor‐like tyrosine kinase; JNK, Jun N‐terminal kinase; RhoA, RAS homolog gene‐family member A; ROCK, Rho‐associated coiled‐coil containing protein kinase; APC, adenomatous polyposis coli; GSK, glycogen synthase kinase 3; LRP5/6, lipoprotein receptor‐related protein‐5/6 receptor; CK1α, Casein kinase. Created with BioRender.com.

The binding of the WNT ligand to lipoprotein receptor‐related protein‐5/6 receptor (LRP5/6) and Frizzled receptor (FZD) on the cell surface inhibits the activity of destruction complex, thereby stabilizing β‐catenin. This leads to the translocation of β‐catenin to the nucleus. In the nucleus, β‐catenin functions as a transcriptional co‐activator of transcription factors belonging to the T‐cell factor (TCF)/ lymphoid enhancer factor (LEF) family. TCF‐LEF regulate embryonic development, stem cells, cell migration, and proliferation (Figure 2, left). Simultaneous binding of R‐Spondin ligands (RSPO1/2/3/4) to ubiquitin‐protein ligases ZNRF3 and RNF43, along with the leucine‐rich‐repeat‐containing G coupled receptors (LGR) 4/5/6, stabilizes FZD receptors for downstream signalling through activation of dishevelled (DSH).

##### Non‐canonical Wnt pathway

The non‐canonical Wnt pathway is β‐catenin independent Wnt pathway, where DKK, a member of the Dickkopf family, is the key player. The binding of DKK to LRP5/6 competitively inhibits the binding of WNT to FZD and LRP5/6 complex. This activates the non‐canonical WNT pathway via the retinoic acid‐related orphan receptors (ROR) and receptor‐like tyrosine kinase (RYK). Activation of Rho‐associated coiled‐coil containing protein kinase (ROCK) and c‐Jun N‐terminal kinase (JNK) signaling aids in maintaining planar cell polarity (PCP) and is one of the underlying mechanisms for the generation of mature epithelial cell‐derived organoids (Figure 2, right).

##### Wnt pathway in GCO development

Wnt signalling pathway decides the fate of the cells, either to go into stemness or planar cell polarity depending on the type of culture conditions present. GCOs can be generated by activating the canonical Wnt pathway. This is achieved by the addition of exogenous WNT3A (known activator of canonical Wnt signaling) either in commercially available pure protein form (Yuan et al., 2022) or collected as a secretory factor from the cell lines (conditioned medium) (Wang et al., 2021). Alternatively, the canonical Wnt pathway is activated by the addition of a commercially available small molecule CHIR99021 (Rimland et al., 2021) that inhibits the GSK3 activity. Inhibition of GSK3 activity prevents phosphorylation and thereby proteasomal degradation of β‐catenin. Stable unphosphorylated β‐catenin molecules then translocate to nucleus to promote expression of TCF/LEF target genes resulting in the canonical Wnt pathway activation.

ASCs isolated from the gallbladder tissue cultured in the presence of a cocktail of growth factors and nutritional supplements maintain the stemness property and drive the fate towards the self‐renewal property. Addition of either exogenous WNT3A or CHIR99021 are required to maintain the self‐renewal property. Cholangiocyte organoids have also been developed from mature cholangiocytes of extrahepatic bile ducts and gallbladder in the presence of DKK‐1, an inhibitor of the canonical Wnt pathway. Organoids developed in the presence of DKK‐1 expressed the key molecular markers of the cholangiocytes and maintained planar cell polarity but lacked expression of the stem cell markers *OCT4, LGR4/5/6, PROM1*, and *NANOG* (Sampaziotis et al., 2017).

Both approaches used R‐Spondin in the culture medium. Thus, R‐Spondin plays an important role in activating both the canonical and the non‐canonical Wnt pathway. However, cholangiocyte organoids have been developed from murine and human tissue even in the absence of R‐Spondin (Scanu et al., 2015) and WNT3A or CHIR99021 (Saito et al., 2019), respectively. GCO was also developed using WNT3A and DKK‐1 in combination (Roos et al., 2021). Thus, GCOs can be developed both by activating or inhibiting the canonical WNT pathways, depending on the signaling cues provided by the culture medium (Online Resource 2).

### Cell source

Cholangiocytes occur at both intrahepatic as well as extrahepatic sites. In the liver, LGR5 is expressed by hepatocytes but not by intrahepatic cholangiocytes (Planas‐Paz et al., 2016). Mouse intrahepatic cholangiocytes appear to be bipotential in nature and under different culture conditions can give rise to hepatocytes or cholangiocytes (Huch et al., 2015). Mouse GCOs express Lgr5 as well as the biliary markers Cldn3, EpCAM, Prom1, Sox17, and Itga6 (Lugli et al., 2016). These techniques were taken forward to develop GCOs from murine tissue (Scanu et al., 2015). GCOs have been successfully developed from murine (Kato et al., 2021; Lugli et al., 2016), bovine (Nagao & Ambrosini, 2023), and porcine (Zarei et al., 2021) tissues. Human intrahepatic cholangiocyte organoids were generated by the addition of forskolin (cyclic AMP pathway agonist), A83‐01 (inhibitor of Transforming growth factor b receptors ALK4/5/7). These protocols were adapted to develop organoids from gallbladder cholangiocytes. GCOs have been developed from surgically resected human gallbladder, mostly from healthy and deceased donors, as well as from malignant ones (Yuan et al., 2022; Wang et al., 2021; Rimland et al., 2021; Sampaziotis et al., 2017; Saito et al., 2019; Roos et al., 2021; Garcia et al., 2020; Sampaziotis et al., 2021; Tan et al., 2022; Zarei et al., 2022; Wang et al., 2022). To the best of our knowledge, there are no reports of developing GCOs from ESCs, PSCs and iPSCs till now.

### Matrix

The extracellular matrix (ECM) plays a pivotal role in organoid development. It provides structural support required for the 3‐D organization. The cell–ECM interactions and mechanical properties (e.g., stiffness, elasticity, and porosity) influences cell behavior, differentiation, proliferation and survival. This in turn regulates tissue polarity and organization. ECM contains a complex mixture of proteins, proteoglycans, and growth factors. These biochemical cues are required to guide cell behavior and fate determination during organoid development.

The most commonly used ECM substitute is derived from solubilized extract from Engelbreth–Holm–Swarm (EHS) murine tumor. For cholangiocyte‐derived organoids both from extra‐hepatic bile ducts and gallbladder, the commonly used matrix is Matrigel. (Kato et al., 2021; Yuan et al., 2022; Wang et al., 2021; Rimland et al., 2021; Sampaziotis et al., 2017; Saito et al., 2019; Roos et al., 2021; Lugli et al., 2016; Zarei et al., 2021; Garcia et al., 2020; Sampaziotis et al., 2021; Tan et al., 2022; Zarei et al., 2022; Wang et al., 2022). Matrigel contains more than 1850 proteins, namely, laminin, collagen IV, nidogen, heparan sulphate proteoglycans, and many other growth factors found in EHS tumors (Hughes et al., 2010). In the presence of suitable temperature and pH these proteins self‐assemble to form 3D fibrous or mesh‐like structures that provide the scaffold for organoid development.

Ideally ECM should be tunable to mimic the specific physicochemical microenvironment of the source tissue. Matrigel sourced from murine sarcoma cells, suffers from batch‐to‐batch variability and cannot be manipulated. Organoids have been established using biologically derived hydrogels like collagen (the most abundant animal protein), laminin (Sato et al., 2009) and basement membrane extract (BME) that provides the scaffold. They have also been grown on synthetic matrices like peptide‐based hydrogels (Treacy et al., 2023) and polyethylene glycol (PEG) based microcavities (Brandenberg et al., 2020). Cholangiocyte‐derived organoids from the bile duct and gallbladder have been developed using densified collagen as the matrix support (Tysoe et al., 2019) and on biologically inert, synthetic and biodegradable scaffold, polyglycolic acid (PGA) (Sampaziotis et al., 2017). These allow such organoids to be used in human organ repair and transplantation.

## APPLICATIONS

The recent developments in the field of organoids developed from healthy organs and tumors are showing promise in research involving disease biology, high throughput drug screening, personalized medicine, tissue engineering, and regenerative medicine. Organoids developed from cholangiocytes isolated from the extrahepatic bile duct or gallbladder have been subjected to various applications.

### Tissue engineering and regenerative medicine

Bile duct disorders such as biliary atresia and ischemic cholangiopathy are difficult to manage and curative option is a liver transplant. Cholangiocyte organoids were propagated on a synthetic biodegradable scaffold made of polyglycolic acid (PGA). PGA is biodegradable, flexible, and also prevents the induction of inflammatory responses in the surrounding regions upon engraftment. Organoids expanded on the PGA scaffolds maintained functionality. Upon transplantation in a healthy immune‐deficient mouse with a surgically damaged gallbladder, these PGA scaffolds populated with the organoids were able to repair the surgical defect in vivo (Sampaziotis et al., 2017).

Primary human cholangiocytes have a transcriptional diversity, not seen in the derived organoids. However, organoids retain the plasticity of the original cholangiocytes when transplanted back into the biliary tree. Utilizing this property, GCOs were perfused into human livers through intraductal delivery (from deceased transplant donors) with significant ischemic duct injury. The organoids engrafted in the intrahepatic biliary tree, expressed key biliary markers with loss of gallbladder markers and regenerated 40%–85% of the damaged ducts (Sampaziotis et al., 2021).

This suggests that in the future readily available cholangiocyte organoids from a biorepository could be used as an adjunct to liver transplant.

### Understanding disease biology

Cholangiocyte organoids can be generated from healthy and diseased biliary tissue, thus allowing researchers to model biliary tract diseases such as cystic fibrosis, cholangitis, cholangiopathy, biliary atresia, gallbladder cancer, and cholangiocarcinoma.

Cholangiocyte organoids have been used to study biliary development by identifying key pathways that are involved during the genesis of normal bile ducts. They were used to model biliary diseases in human such as Alagille syndrome and cystic fibrosis. Normal organoids have a functional cystic fibrosis transmembrane conductance regulator (CFTR). Cholangiocyte organoids derived from patients with cystic fibrosis have functionally impaired CFTR and this was corrected when organoids were exposed to chemical correctors (Ogawa et al., 2015). In another cholangiocyte organoid model of cystic fibrosis, an experimental drug was shown to rescue the phenotype (Sampaziotis et al., 2015).

**FIGURE 3:**
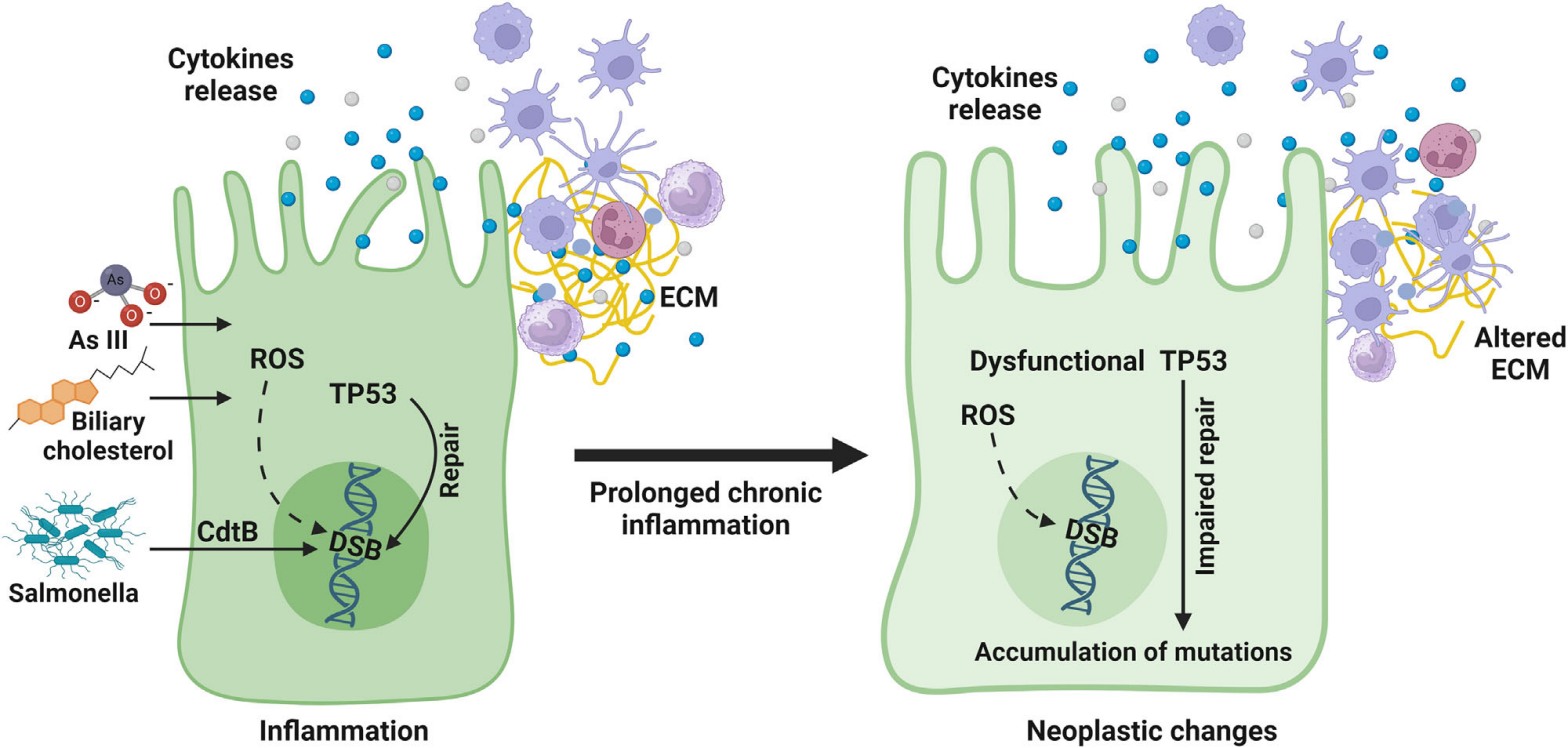
**Schematic diagram of gallbladder cancer pathogenesis**. Metabolic (Larsson & Wolk, 2007) and environmental stress (Ganesan et al., 2020) induced ROS generation and/or bacterial toxin CdtB result in DNA double strand breaks. In presence of functional TP53 and DNA repair system, DNA damage is repaired. Continuous exposure to oxidative stress causes repeated cycles of DNA damage and repair, resulting in prolonged chronic inflammatory condition. This ultimately results in dysfunctional DNA repair system leading to accumulation of mutational burden and thus oncogenic transformation of gallbladder cholangiocytes (Roa et al., 2022). CdtB, cytolethal distending toxin subunit B; DSB, double‐strand breaks; ROS, reactive oxygen species; ECM, extracellular matrix; As III, trivalent inorganic arsenic ions. Created with BioRender.com.

The most likely etiology for GBC is chronic inflammation resulting in a DNA damage response and an associated APOBEC 2/13 mutation signature (Alexandrov et al., 2013) (Figure 3). Chronic inflammation leads to bile inspissation and stasis predisposing to gallstones. Gallbladder columnar epithelia are protected from alkaline bile by prostaglandin E2 (PGE2) induced mucin secreted by goblet cells, a process regulated by COX2. Obesity, diabetes and female sex increase cholesterol excretion into the bile. Decreased emptying of the gallbladder results in increased concentrations of cholesterol and hydrophobic bile salts, increasing oxidative stress (generation of reactive oxygen species), decreased bile pH and initiates an inflammatory process. Macrophages uptake cholesterol diffusing through the gallbladder, potentially initiating chronic metabolic inflammation. Polymorphisms in *ABCB4* are associated with impaired phospholipid transport into bile and development of gallstones and in *ABCB1* with inflammatory bowel disease. The majority (>90%) of GBC cases are adenocarcinomas. Of the ∼1200 mutated genes identified, nonsynonymous mutations of *TP53 (mutTP53)*, often with loss of heterozygosity (LOH) of 17p, are most frequent. Activating *KRAS* mutations seen in 18% of Japanese (Nakamura et al., 2015). Chinese and Japanese studies suggest a type 2/13 mutational signature characteristic of APOBEC mediated mutations (Alexandrov et al., 2013). Activating mutations of genes in the ERBB signaling pathway (∼8%) are reported in Chinese and Indian patients. Mutations in chromatin remodeling genes (e.g., *BAP1, ARID1/2, PBMR1*) described in other studies (Nakamura et al., 2015), were not identified in Chinese patients. No detailed analyses of Indian patients have been performed. A multistage evolution model of GBC has been proposed based on the results of these investigations. Pre‐cancerous changes include *TP53* mutations, COX2 overexpression, mitochondrial DNA mutations, and abnormal hypermethylation of promoters of various tumor‐suppressor genes. Towards this CRISPR‐Cas9 deletion of *Trp53* was performed on GB organoids from *Kras^LSL‐G12D/+^* mice of the C57BL/6J strain. When transplanted subcutaneously into syngeneic competent mice, the organoids developed tumors and immune microenvironment similar to that observed in patients with GBC (Kato et al., 2021).

*Salmonella* species are known to colonize the gallbladder. *Salmonella* produce human specific cytolethal distending toxin (Cdt). CdtB subunit has DNase I like activity and induces DNA double‐strand breaks (DSB) with activation of the ataxia‐telangiectasia mutated (ATM)–CHK2 signaling pathway and phosphorylation of histone H2AX (Gunn et al., 2014). *Salmonella typhimurium* induced malignant transformation in a murine *Trp53* mutated and *c‐Myc* amplified GCO model (Scanu et al., 2015). In a human GCO model, *Salmonella enterica* were able to invade the epithelial cells of the organoid and induce DNA DSB and a cell cycle arrest. The CdtB subunit transferred to neighboring uninfected cells and induced DSB, inducing genomic instability. Thus, GCOs serve as a model to study the host‐pathogen interactions.

We have previously reported that patients with GBC mostly present with advanced disease and many have had an earlier cholecystectomy with an incidental diagnosis of GBC (Dutta et al., 2023). This means that there is often limited surgical material. Thus, cholangiocyte organoids have usually been more successfully generated from other biliary tract cancers (Saito et al., 2019). Nevertheless, a number of groups have successfully generated malignant GCO which have been maintained through repeated passages and have been successfully cryopreserved (Yuan et al., 2022; Wang et al., 2021; Saito et al., 2019; Garcia et al., 2020). GCO from both benign and malignant tissue have also been successfully generated from bile samples obtained at endoscopic retrograde cholangiopancreatography (Kinoshita et al., 2023), with a combination of repeated passage, xenograft and Nutlin‐3a treatment to minimize the growth of normal cells. In general, malignant GCO retain the histological and genetic characteristics of the original tumor and can be used to create xenografts. Comparative transcriptomic analyses of GCOs derived from normal, benign adenomas and adenocarcinoma show progressive upregulation of cell cycle‐ and DNA replication‐related pathways and downregulation of p53 signalling and extracellular matrix (ECM)‐receptor interaction pathways in GBC organoids (Yuan et al., 2022), suggesting that GCO generated from different GB pathologies will be suitable models to investigate the pathogenesis of GBC.

### Drug screening

Cholangiocyte organoids provide a platform for screening potential therapeutics for biliary diseases (Table 1). The number of studies using cholangiocyte organoid based drug screening for gallbladder or biliary tract cancers are limited. Nevertheless, drug screening studies show that the majority of the malignant cholangiocyte organoid lines respond to gemcitabine, the drug most commonly used in clinic to treat GBC. In addition, the studies have identified potential alternative targets for GBC and biliary tract cancers. Gemcitabine‐resistant GBC patients had upregulation of yes‐associated protein‐1 (YAP1). YAP1 inhibition (by targeting the Hippo pathway) in the malignant organoids sensitize the drug tolerant tumor cells to gemcitabine (Garcia et al., 2020). In gemcitabine‐refractory biliary tract cancer organoids, treatment with microRNA‐451a (miR‐451a) inhibited the cancer progression by abrogating the Phosphoinositide 3‐kinase/ Akt kinase (PI3K/Akt) pathway (Obata et al., 2023). Another study shows histone deacetylase (HDAC) inhibitors or dual PI3K/HDAC inhibitors restricted the growth of GBC organoids developed from patients where HDAC1, 2 and 6 are overexpressed without causing toxicity to normal GCOs (Yuan et al., 2022). The combinatorial treatment of gemcitabine with 5‐FU or cisplatin and 5‐FU with trastuzumab elicited a better tumor suppression compared to the drugs alone. A co‐culture system of GBC organoids and peripheral blood mononuclear cells identified the immune checkpoint inhibitor nivolumab as a potential therapeutic for GBC and this was confirmed in humanized mouse model (Tan et al., 2022). Only two studies so far have directly tested the clinical correlation of the drug sensitivity of GBC or cholangiocarcinoma organoids at the personalized level that too for only three (Wang et al., 2021) and one (Tan et al., 2022) patients, respectively. Larger cohort studies are required for true assessment of the predictive value of GCO based assays for clinical response.

**TABLE 1:** Patient‐derived biliary tract and gallbladder cancer organoids for drug response study.

Cancer type	Drugs and dose tested	Treatment duration	Assay to detect drug response	Key outcomes	Reference
BTC	Compound library of 339 clinically approved drugs – 0.1 µM final concentration	6 days	WST	Antifungals as potential therapeutics for BTCs	(Saito et al., 2019)
GBC	Gemcitabine –10 µM to 0.2 nM alone and also in combination with Verteporfin (IC90 dose of both)	3 days	CTG	YAP1 as a potential candidate for targeted therapy	(Garcia et al., 2020)
Extrahepatic BTC	Gemcitabine, 5‐ fluorouracil, cisplatin, paclitaxel, infigratinib or ivosidenib – 0.1, 1, 10, 100 and 500µM	4 days	CTG	Gemcitabine found to be most efficient. Mixed results in clinical correlation for three patients.	(Wang et al., 2021)
GBC	29 compounds approved for clinical use	4 days	CTG	Dual PI3K/HDAC inhibitor suppressed the growth of GBC organoids without affecting the normal GCOs	(Yuan et al., 2022)
GBC	Gemcitabine—(9.6 nM to 30 µM) Irinotecan—(0.944 nM to 2.95 µM) Cisplatin—(0.8544 nM to 2.67 µM) 5‐fluorouracil—(3.2 nM to 10 µM) Herceptin & Nivolumab‐ (32 ng/ml to 100 µg/ml) alone and in combination	1 day	CTG	Combinatorial treatment of gemcitabine and cisplatin with nivolumab suppressed tumor growth. Clinically correlated in one patient.	(Tan et al., 2022)

Abbreviations: BTC, Biliary tract cancer; CTG, CellTiter‐Glo3D cell viability assay; GBC, Gallbladder cancer; WST, Water‐soluble tetrazolium salts assay.

## LIMITATIONS AND FUTURE SCOPE

Despite the potential that organoid models hold for various applications, its broad utility is yet to be realized. Like every study model, organoid model has its own share of limitations and GCO is no exception.

Epithelial organoids like GCO models are developed only from single cell type. Whereas, in tissues there is coexistence and cross‐talk among various component cell types including immune cells, fibroblasts and other stromal cells with epithelial cells. Besides, absence of vasculature limits the recapitulation of the tissue‐vascular endothelia interactions as well as exchange of metabolites, nutrients, gases, and drugs between organs and body fluid. Thus, lack of complete reconstitution of the tissue microenvironment (TME) is an inherent limitation of the organoid model system. Co‐culturing of fibroblasts, immune cells or other stromal cells with epithelial organoids are some of the approaches taken to mimic the multicellular cross‐talks. These cross‐talks modulate tumor growth and drug response (Yuan et al., 2022). Modelling the immune cell interactions in organoid culture is of particular importance for testing the drug responses that require involvement of the immune system. For example, GCOs developed from GBC responded to nivolumab, an immune checkpoint inhibitor, only when co‐cultured with peripheral blood mono nuclear cells (Tan et al., 2022). Air‐liquid interface (ALI) based organoid culture system has been used incorporating intestinal epithelial cells and mesenchymal/stromal cells (Li et al., 2016). ALI based culture systems could also be used to study the tumor‐immune system interactions keeping the endogenous stromal components of the native TME (Yuki et al., 2020). Applications of microfluidics have been tested in organ‐on‐chip systems to model the body fluid circulation for different organoid models, but no report yet available for GCOs. Finally, unlike the in vivo model systems, organoids exist in isolation from the multi‐organ system. While multi‐organ organoid system is a complex model to develop, hepato‐biliary‐pancreatic organoid systems have been developed using human pluripotent stem cells (Koike et al., 2019). This type of multi‐organ organoid systems involving gallbladder organoid could be useful in studying disease pathogenesis at the biliary system level.

Murine EHS derived ECM, which is the matrix most commonly used for GCO development or organoid culture in general, suffers from lot‐to‐lot variability. Presence of murine proteins in the matrix risk interference with the outcome of the patient derived organoid based assays. Besides, for applications in regenerative medicine, it is essential to use a matrix system, that is, non‐immunogenic and biodegradable. Xeno‐free, defined compositions such as synthetic peptide‐based hydrogels optimized for organoid development could be an alternative (Treacy et al., 2023). Stiffness and rheological properties of the matrix varies depending on the types of tissue and pathological conditions. For example, stiffness (elastic modulus) of healthy gallbladder tissues have been reported to be in the range of 255 + 24.55 kPa (axial) between 641.2 + 28.12 kPa (transverse) (Karimi et al., 2017). Whereas, malignant gallbladder tissues have higher stiffness (Vijayakumar et al., 2013). Ideal matrix for GCO should be tunable to match the mechano‐chemical properties of the gallbladder tissues of different pathological conditions. Other sources of variability could originate from the alterations in organoid growth media compositions used by different research groups (Online Resource 2). Culture media components can potentially modify the degree of differentiation, morphology and behavior of the organoids. Variations in culture media may also influence the extent of clonal heterogeneity preserved in the tumor GCOs.

All the above factors can contribute to reduced reproducibility and loss of clinical relevance. Like any pre‐clinical models, developed GCOs for each study should be carefully validated at the histological, molecular, and functional level. Lack of well annotated GCO models and well‐designed large cohort studies have limited the assessment of true potential of GCO as a pre‐clinical model.

Finally, for robust adoption of GCOs and other organoid based high throughput assays, limited clinical resources and expensive culture reagents are two bottlenecks. As availability of surgically resected GBC is often a challenge, primary cells can be availed through alternative procedures such as endoscopic retrograde cholangiopancreatography (ERCP), needle core biopsy, and fine needle aspirations. While there are no reports of successful GCO developed from the latter two methods, cholangiocyte organoids have been successfully developed from ERCP brush cells (Tysoe et al., 2019). To make the organoid assays affordable, it is imperative to scale down organoid culture to 384‐ or 1536‐well culture dish systems. At this scale, automation is essential for reproducibility. Application of 3D bioprinting, advanced microscopy such as light sheet fluorescence or high‐content micro confocal imaging along with AI/ML based imaging analysis would enable GCO based high throughput assays to identify alternative therapeutics for gallbladder cancer.

## AUTHOR CONTRIBUTIONS

Conceptualization: VS; Literature Search: AD, PG, NC, SC; Draft preparation: AD, NC, DGS; Critical Revision: DGS and VS; Final version reading and approval: All.

## CONFLICT OF INTEREST STATEMENT

Vaskar Saha acknowledges receiving a research grant for a joint research project (unrelated to this study) from Gennova Biopharmaceuticals Ltd. The funding sources have no role in the design, practice or analysis of this study. Other authors have no conflicts of interest to disclose.

## Supporting information



Supporting Information

Supporting Information

## Data Availability

Data sharing not applicable to this article as no datasets were generated or analyzed during the current study.
