# Supplementary material for: Gallbladder cholangiocyte organoids
Source: Biol Cell. 2025 Feb 13;117(2):e2400132. doi: 10.1111/boc.202400132 (PMC11823593; doi:10.1111/boc.202400132)
Supplement: Supplementary file 1 — Supporting Information [file BOC-117-e2400132-s001.docx]

**Title of Review Article: Gallbladder cholangiocyte organoids**

**Journal name: Biology of the Cell**

Ankita Dutta^1,2^, Nandita Chowdhury^1^, Shinjini Chandra^1^, Payel Guha^1^, Vaskar Saha^1,3,4, ✉^, Dwijit GuhaSarkar^1^

^1^ SOLi3D Laboratory, Tata Translational Cancer Research Centre, Kolkata, India,

^2^ School of Medical Science and Technology, Indian Institute of Technology, Kharagpur, India,

^3^ Department of Paediatric Haematology and Oncology Tata Medical Center, Kolkata, India,

^4^ Division of Cancer Sciences, Faculty of Biology, Medicine and Health, School of Medical Sciences, University of Manchester, Manchester, UK University of Manchester, Manchester, UK

✉Correspondence to:

Vaskar Saha

Tata Translational Cancer Research Centre, Tata Medical Center, MAR 14 (E-W), Newtown, Kolkata 700160, India

Email: [v.saha@manchester.ac.uk](mailto:v.saha@manchester.ac.uk)

Telephone (office): 033-6605-7810

**Supplementary Table 1. Different types of organoids with their cell sources from human and their application**

| **Tissue or organ of origin** | **Source** | **Application** | **References** |
| --- | --- | --- | --- |
| Cerebral structure | PSCs | Transplantation in mouse restored sensorimotor function after stroke | [1] |
| Brain | ESCs | Understanding brain development and modelling brain tumour | [2] |
| Salivary gland | ASCs | Understanding the characteristics of the salivary gland carcinoma subtypes | [3] |
| Mammary gland | ASCs | Drug screening of patient derived and genetically modified organoids | [4] |
| Thyroid gland | ASCs | Investigating tumour biology and drug screening | [5] |
| Stomach | ASCs | Drug screening | [6] |
| Liver | ASCs | Disease modelling and drug screening | [7] |
| Gallbladder | ASCs | Modelling host-pathogen (*Salmonella typhi)* interaction in gallbladder | [8] |
|  | ASCs | Drug response testing in gallbladder cancer | [9] |
|  | Mature epithelial cells | *Ex vivo* modelling of gallbladder in human | [10] |
| Pancreas | ASCs | Pancreatic cancer modelling and personalised drug screening | [11] |
| Small intestine | PSCs | Modelling for molecular pathogenesis of gut disease | [12] |
|  | ASCs | Modelling the intestinal development, homeostasis and tissue repair | [13] |
| Colon | ASCs | High content drug screening and drug repurposing | [14] |
|  | PSCs | Recapitulating the colon microenvironment, thus enabling immune based therapy in future | [15] |
| Prostate | ASCs | A platform for drug screen and advancement in personalized medicine | [16] |
| Fallopian tube | ASCs | Understanding fallopian tissue renewal and role in disease progression | [17] |
| Kidney | ESCs | Modelling healthy and diseased kidney to understand nephrogenesis | [18] |

**References for Supplementary Table 1**

1. Zhou T, Tan L, Cederquist GY, et al. High-Content Screening in hPSC-Neural Progenitors Identifies Drug Candidates that Inhibit Zika Virus Infection in Fetal-like Organoids and Adult Brain. Cell Stem Cell. 2017;21:274-83 e5.

2. Hendriks D, Pagliaro A, Andreatta F, et al. Human fetal brain self-organizes into long-term expanding organoids. Cell. 2024;187:712-32 e38.

3. Aizawa Y, Takada K, Aoyama J, et al. Establishment of experimental salivary gland cancer models using organoid culture and patient-derived xenografting. Cell Oncol (Dordr). 2023;46:409-21.

4. Sachs N, de Ligt J, Kopper O, et al. A Living Biobank of Breast Cancer Organoids Captures Disease Heterogeneity. Cell. 2018;172:373-86 e10.

5. Chen D, Su X, Zhu L, et al. Papillary thyroid cancer organoids harboring BRAF(V600E) mutation reveal potentially beneficial effects of BRAF inhibitor-based combination therapies. J Transl Med. 2023;21:9.

6. Xu J, Gong J, Li M, et al. Gastric cancer patient-derived organoids model for the therapeutic drug screening. Biochim Biophys Acta Gen Subj. 2024;1868:130566.

7. Broutier L, Mastrogiovanni G, Verstegen MM, et al. Human primary liver cancer-derived organoid cultures for disease modeling and drug screening. Nat Med. 2017;23:1424-35.

8. Sepe LP, Hartl K, Iftekhar A, et al. Genotoxic Effect of Salmonella Paratyphi A Infection on Human Primary Gallbladder Cells. mBio. 2020;11.

9. Yuan B, Zhao X, Wang X, et al. Patient-derived organoids for personalized gallbladder cancer modelling and drug screening. Clin Transl Med. 2022;12:e678.

10. Tysoe OC, Justin AW, Brevini T, et al. Isolation and propagation of primary human cholangiocyte organoids for the generation of bioengineered biliary tissue. Nat Protoc. 2019;14:1884-925.

11. Driehuis E, van Hoeck A, Moore K, et al. Pancreatic cancer organoids recapitulate disease and allow personalized drug screening. Proc Natl Acad Sci U S A. 2019.

12. Kwon O, Lee H, Jung J, et al. Chemically-defined and scalable culture system for intestinal stem cells derived from human intestinal organoids. Nat Commun. 2024;15:799.

13. Gjorevski N, Sachs N, Manfrin A, et al. Designer matrices for intestinal stem cell and organoid culture. Nature. 2016;539:560-4.

14. Mertens S, Huismans MA, Verissimo CS, et al. Drug-repurposing screen on patient-derived organoids identifies therapy-induced vulnerability in KRAS-mutant colon cancer. Cell Rep. 2023;42:112324.

15. Munera JO, Kechele DO, Bouffi C, et al. Development of functional resident macrophages in human pluripotent stem cell-derived colonic organoids and human fetal colon. Cell Stem Cell. 2023;30:1434-51 e9.

16. Karkampouna S, La Manna F, Benjak A, et al. Patient-derived xenografts and organoids model therapy response in prostate cancer. Nat Commun. 2021;12:1117.

17. Kessler M, Hoffmann K, Brinkmann V, et al. The Notch and Wnt pathways regulate stemness and differentiation in human fallopian tube organoids. Nat Commun. 2015;6:8989.

18. Ungricht R, Guibbal L, Lasbennes MC, et al. Genome-wide screening in human kidney organoids identifies developmental and disease-related aspects of nephrogenesis. Cell Stem Cell. 2022;29:160-75 e7.
